# Supplementary material for: Development and Validation of a Reliable UHPLC-MS/MS Method for Simultaneous Quantification of Macrocyclic Lactones in Bovine Plasma
Source: Molecules. 2022 Feb 1;27(3):998. doi: 10.3390/molecules27030998 (PMC8838099; doi:10.3390/molecules27030998)
Supplement: Supplementary file 1 [file molecules-27-00998-s001.zip › molecules-1551301-supplementary.pdf]

**Appendix A**

**Supplementary figures and tables**

**Table S1:** Evaluation of extraction recovery of standard solutions of 25ng/ml of ivermectin (IVER), doramectin (DORA) and moxidectin (MOXI) in water (250 µl)/extraction solvent (750 µl) after pass-through on an Oasis® PRiME 96-well µ-Elution plate and OstroTM 96-well plate.

| SPE Method | Extraction solvent | Extraction recovery (%) | | |
|---|---|---|---|---|
| | | IVER | MOXI | DORA |
| PRiME | 1 % FA in ACN | 63.6 | 57.3 | 70.7 |
| | 1% FA in MeOH | 0.1 | 1.2 | 0.1 |
| | 1% FA in EtOH | 58.3 | 22.4 | 55.6 |
| Ostro™ | 1 % FA in ACN | 84.8 | 98.1 | 97.0 |
| | 1% FA in MeOH | 36.4 | 54.7 | 61.8 |
| | 1% FA in EtOH | 88.0 | 74.8 | 92.6 |

Note: FA = formic acid, ACN = acetonitrile, MeOH = methanol, EtOH = ethanol

(A)

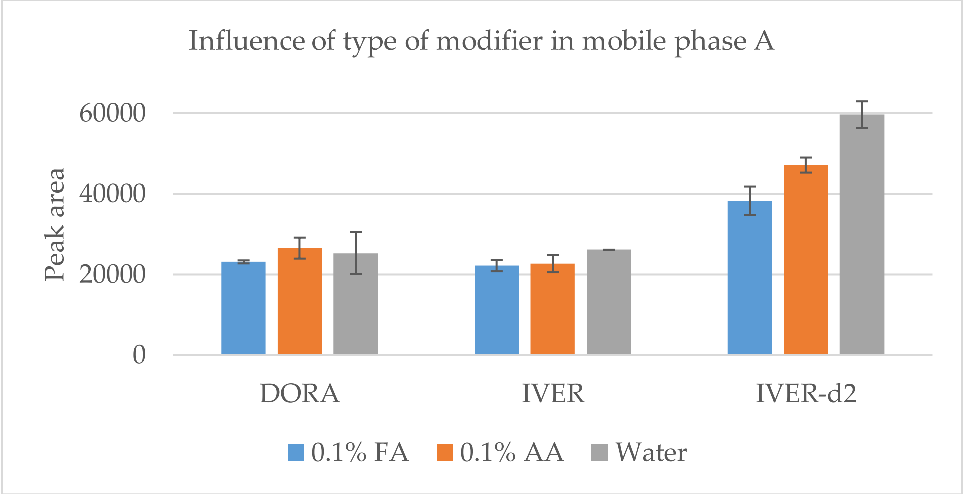

(B)

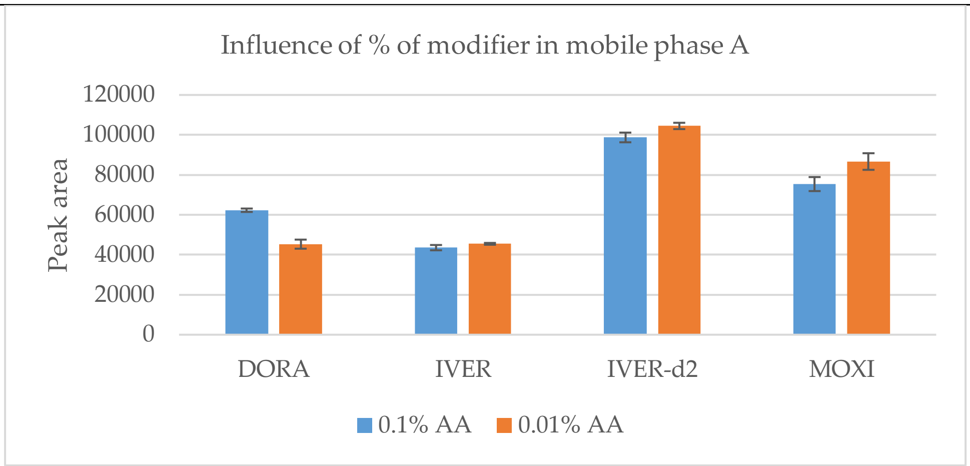


(C)

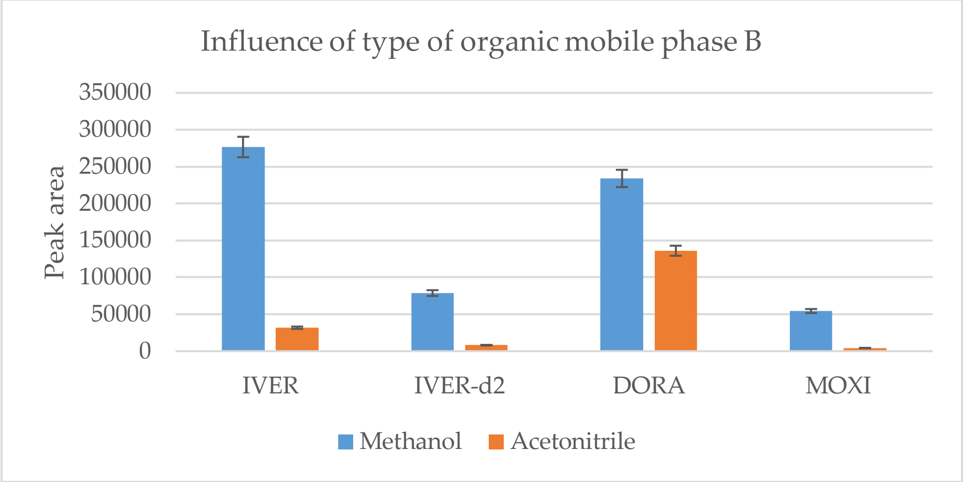


**Figure S1:** Influence of mobile phase composition on the peak area of each analyte in 100 ng/ml standard solutions (injection volume: 10 µl): (A) mobile phase A: 0.1 % formic acid (FA) in water, 0.1 % acetic acid (AA) in water or water without modifier; mobile phase B: acetonitrile; (B) mobile phase A: 0.1 % AA in water or 0.01 % AA in water, mobile phase B: acetonitrile; (C) mobile phase A: 0.01 % AA in water, mobile phase B: acetonitrile or methanol.

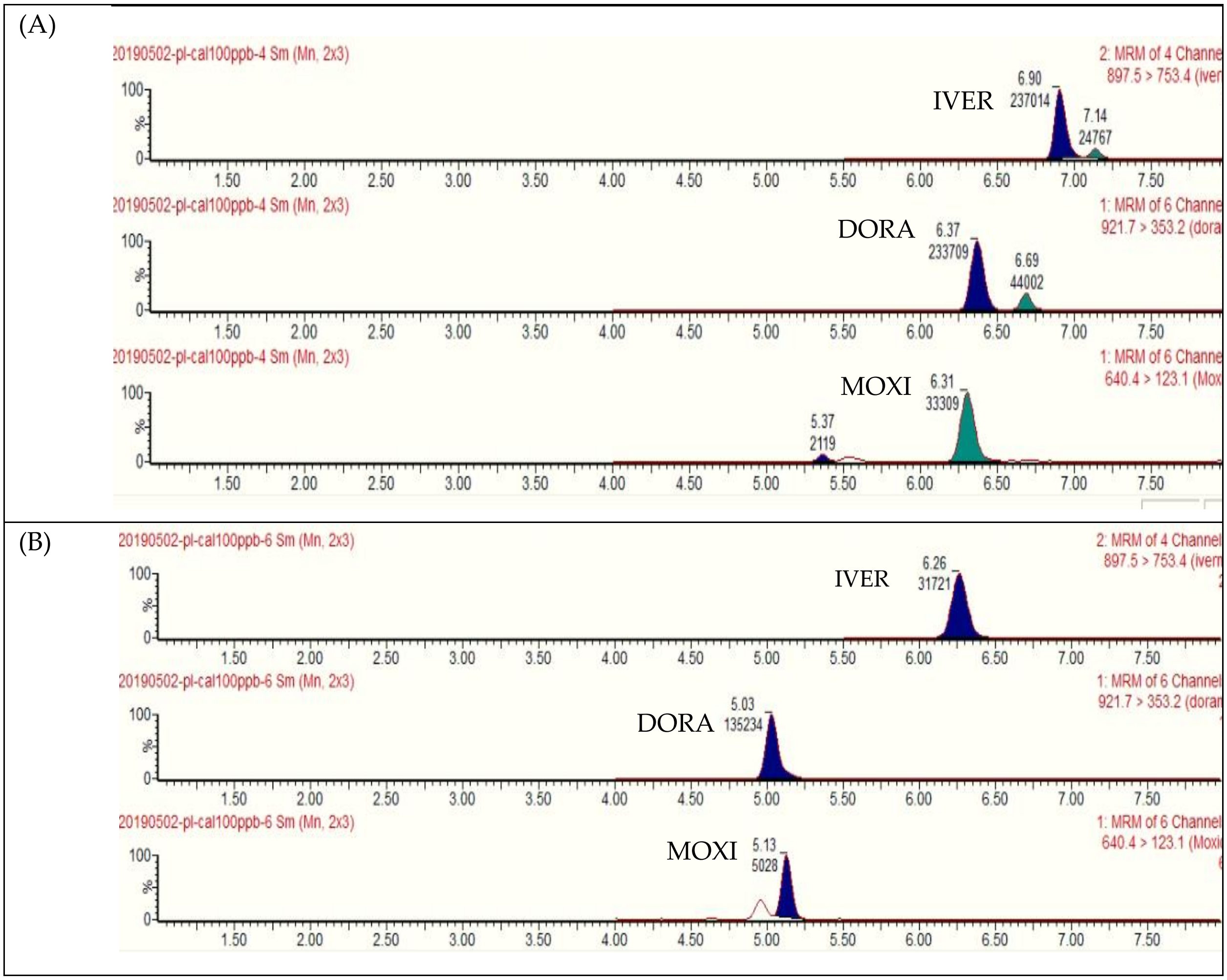


**Figure S2:** MS/MS chromatogram of a spiked plasma sample (analyte concentration: 100 ng/ml) that was analyzed using 0.01 % AA in water as mobile phase A and 0.01 % AA in methanol (panel A) or 0.01 % AA in acetonitrile (panel B) as mobile phase B. The following gradient program was applied: 0 – 0.5 min: 20 % A/80 % B; 0.5 – 6.0 min: linear gradient to 99 % B; 6.0 – 7.5 min: 1 % A/99 % B; 7.5 – 7.7 min: linear gradient to 80 % B; 7.7 – 10.0 min: 20 % A/80 % B.
